# Conceptual and Disclosure Considerations in Applying Neuroaesthetics to Aesthetic Medicine

**DOI:** 10.1111/jocd.71061

**Published:** 2026-07-08

**Authors:** Steven Harris, Alain Michon

**Affiliations:** ^1^ Harris Clinic London UK; ^2^ Project Skin Ottawa Canada

To the Editor,

We read with interest the recent narrative synthesis by Dayan and Fabi proposing neuroaesthetics as a framework for aesthetic medicine [1]. The thesis is ambitious: that perceptions of naturalness and attractiveness are mediated by neural systems and should therefore anchor clinical practice. There is much in the underlying intuition that warrants thoughtful consideration. We offer several observations regarding the article's conceptual framing, together with one issue of disclosure transparency that may benefit from further clarification.

It is worth stating at the outset what the framework gets right. The neuroaesthetic tradition the authors invoke rests on a substantial body of work: functional imaging has identified reasonably consistent neural correlates of aesthetic appreciation, and the perception of facial attractiveness has been linked to the processing of averageness, symmetry, and configural relationships across numerous studies [2, 3]. The authors are correct that observer perception is neurally mediated, and that this dimension has been under‐theorized in a field often preoccupied with anatomy and product alone. Our concern is not with this premise, but with how far the present paper extends it and on what evidential basis.

The article is presented as a “narrative synthesis of interdisciplinary literature,” yet no search strategy, no inclusion or exclusion criteria, and no method for weighting evidence are reported. Without these features, readers cannot distinguish systematic appraisal from selective citation. The article may be more accurately characterized as a conceptual commentary than a formally structured narrative synthesis.

Several central conceptual claims rely on preliminary, contested, or limited evidence. While some of the perceptual findings the authors cite are well established, others are not. The Zajonc vascular theory of emotion, invoked to suggest that overfilling the deep medial cheek may alter brain temperature and mood, has been subject to substantial criticism and has not achieved broad empirical support. The Sakano fMRI study on skin reflectance is a small psychophysical experiment, generalized here into sweeping claims about “neural activation.” A pilot observation on visually impaired individuals “detecting beauty” is flagged by the authors as preliminary yet is subsequently used to support broader claims regarding subconscious aesthetic perception.

The proposed framework also struggles with falsifiability. A natural‐looking outcome confirms neuroaesthetic principles. An unnatural one also confirms them, by way of the uncanny valley or a failed peak‐shift. A patient who feels better confirms the model; one who does not is explained through a different sub‐loop. A framework capable of accommodating almost any outcome risks becoming difficult to falsify in practice, and theories that predict everything ultimately predict very little.

The paper's critique of formulaic injection approaches may also overstate how experienced injectors currently approach facial assessment and treatment planning. Serious practitioners have written extensively against algorithmic injection, including critiques of what has been described as “aesthetic dumbification.” [4] The authors then offer their own six‐step “feedback model” and standardized consultation script as the corrective. Replacing one structured framework with another, albeit reframed through neuroscientific terminology, may not represent the paradigm shift implied in the abstract.

Finally, the term itself carries weight. Neuroaesthetics, as developed by Zeki, Chatterjee, and colleagues, is a rigorous research program concerned with how the brain processes aesthetic experience across art, music, and form [2, 3]. Its application to aesthetic medicine warrants conceptual care to avoid diluting what the field has come to mean.

None of this dismisses the underlying point. Naturalness, dynamic expression, identity preservation, and observer perception are genuinely important and under‐theorized dimensions of aesthetic outcome. The neural basis of some of these is reasonably well supported; for example, the consistent involvement of reward circuitry in the appreciation of attractive faces, the role of averageness and symmetry as perceptual cues, and the authors' broader contributions to this conversation are well known. But the paper, in our view, conflates an important clinical sensibility with a broader scientific framework that remains insufficiently established.

Importantly, this does not preclude future empirical work from operationalizing and rigorously testing neuroaesthetic hypotheses within aesthetic medicine. The intuition is worth the effort of formalization; our argument is that the formalization has not yet been done.

A final observation concerns disclosure. Both authors are associated with XOMD Skin, a topical formulation marketed within the “Moodceuticals”/“neurocosmetics” category, the clinical validation for which was published in this same journal [5, 6] under conceptual language, the “psycho‐social‐dermal axis,” closely paralleling the “skin–brain–social axis” the present paper now theorizes. Given the conceptual overlap between the framework proposed here and a previously published commercial‐research program, readers may benefit from a more specific disclosure, consistent with ICMJE recommendations on financial relationships involving closely related products [7].

Aesthetic medicine is at a point where its claims to scientific seriousness are increasingly tested by ultrasound, rigorous outcome measurement, and external scrutiny. We offer these observations in that spirit and respectfully suggest that a more specific Conflict of Interest disclosure would aid readers in interpreting the work.

Yours sincerely,

## Author Contributions

Both authors contributed to the conception, drafting, and revision of this correspondence and approved the final version for submission.

## Funding

The authors have nothing to report.

## Conflicts of Interest

Dr Harris declares no conflicts of interest. Dr. Alain Michon serves as an Associate Editor of the Journal of Cosmetic Dermatology. He played no editorial role in the handling, peer review, or acceptance of the article to which this correspondence refers, and has recused himself from any editorial consideration of this letter. He is also a consultant for Allergan Aesthetics and SkinCeuticals. The authors otherwise declare no commercial interests relevant to the subject matter of this correspondence.

## Data Availability

The authors have nothing to report.
